# Glucocorticoids and endothelial cell barrier function

**DOI:** 10.1007/s00441-013-1762-z

**Published:** 2013-12-19

**Authors:** Ellaine Salvador, Sergey Shityakov, Carola Förster

**Affiliations:** Department of Anesthesia and Critical Care, Center for Operative Medicine, University Hospital Würzburg, Würzburg, Germany

**Keywords:** Glucocorticoids, Glucocorticoid receptor, Blood brain barrier, Endothelial cells

## Abstract

Glucocorticoids (GCs) are steroid hormones that have inflammatory and immunosuppressive effects on a wide variety of cells. They are used as therapy for inflammatory disease and as a common agent against edema. The blood brain barrier (BBB), comprising microvascular endothelial cells, serves as a permeability screen between the blood and the brain. As such, it maintains homeostasis of the central nervous system (CNS). In many CNS disorders, BBB integrity is compromised. GC treatment has been demonstrated to improve the tightness of the BBB. The responses and effects of GCs are mediated by the ubiquitous GC receptor (GR). Ligand-bound GR recognizes and binds to the GC response element located within the promoter region of target genes. Transactivation of certain target genes leads to improved barrier properties of endothelial cells. In this review, we deal with the role of GCs in endothelial cell barrier function. First, we describe the mechanisms of GC action at the molecular level. Next, we discuss the regulation of the BBB by GCs, with emphasis on genes targeted by GCs such as occludin, claudins and VE-cadherin. Finally, we present currently available GC therapeutic strategies and their limitations.

## Introduction

Glucocorticoids (GCs) belong to a class of steroid hormones that bind to GC receptors (GRs), which, once activated as a complex, upregulate the expression of anti-inflammatory proteins in the nucleus and repress the expression of proinflammatory proteins in the cytosol (Rhen and Cidlowski 2005). GCs are the first-choice therapy for inflammatory disease and a common anti-edematous agent. Administration of GCs is employed as a novel treatment strategy for several central nervous system (CNS) diseases such as brain tumors, brain edema and multiple sclerosis (MS).

 The neurovascular unit of the CNS is made up of microvascular endothelial cells that seal the paracellular spaces between the blood and brain and, thus, are often referred to as the blood brain barrier (BBB), together with circulating blood components, pericytes, astrocytes and neurons (Neuwelt et al. 2008). In many disorders of the CNS, the integrity of the BBB is compromised. Since GCs are found to improve barrier properties, they are often used for the therapy of CNS disorders that involve the BBB. GC treatment has demonstrated a tightening of the BBB in the murine microvascular brain endothelial cells, cEND (Förster et al. 2005, 2006).

## Mechanisms of GC action at the molecular level

**Fig. 1:**
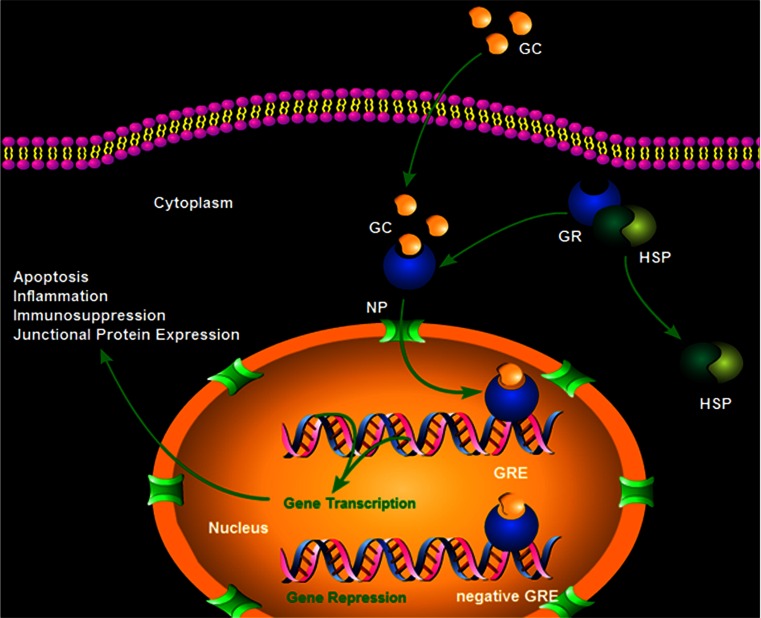
Mechanisms of glucocorticoid (*GC*) action. GC crosses the cell membrane and binds to the glucocorticoid receptor (*GR*) in the cytoplasm. GRs are kept in an inactive state and prevented from moving into the nucleus by heat shock proteins (*HSP*). Upon binding of a GC to a GR, the HSP dissociates and the GC-GR complex moves to the nucleus via a nuclear pore (*NP*). The complex then binds to GC-responsive elements (*GRE*) in the 5′ promoter region of DNA. Transcription is then activated. However, if the region contains a negative GRE, transcription is repressed

GCs act on a wide variety of cell types leading to many different physiological and pathological responses and systemic effects. These responses and effects are mostly attributable to the ubiquitous nature of the GR. The action of GCs begins when the GC crosses the cell membrane and binds to a GR (Fig. 1). GRs are located in the cytoplasm and belong to the thyroid/retinoic acid receptor superfamily, which is composed of ligand-dependent transcription factors (Evans 1988; Tsai and O’Malley 1995). Within the cytoplasm, GRs are maintained in an inactive state by being bound to heat shock proteins (hsps) to prevent them from moving into the nucleus (Wikstrom et al. 1986; Picard et al. 1988; Dao-Phan et al. 1997). Once the GC binds to a GR, the hsps dissociate and the GC-GR complex then translocates into the nucleus in which it dimerizes with a second GC-GR complex (Dittmar et al. 1997; Hawle et al. 2006).

The zinc-finger domains of the GR can bind to specific DNA sequences, i.e., GC-responsive elements (GREs), in the 5′ promoter region. The GR can then activate transcription in a process termed GR transactivation (Beato 1989; Schaaf and Cidlowski 2003; Harke et al. 2008). However, if the region contains a negative GRE, transcription is repressed (Sakai et al. 1988; Drouin et al. 1989; Nakai et al. 1991; Subramaniam et al. 1998).

The inflammatory and immunosuppressive effects of GCs rely on several molecular mechanisms that include (1) direct effects on gene expression by the binding of GRs to GREs; (2) indirect effects on gene expression by the interaction of GRs with other transcription factors; (3) GR-mediated effects on second messenger cascades, i.e., the P13K-Akt-eNOS pathway (Rhen and Cidlowski 2005).

## Glucocorticoid receptor

GRs mediate the biological effects of GCs (Beato 1989). They are a type II steroid receptor and transcription factor and alter the expression of target genes involved in multiple cellular processes such as immune function, inflammation and cell death (Schaaf and Cidlowski 2003). As a type II receptor, GR is expressed in almost all tissues and cells. It has a binding affinity for cortisol of approximately 30 nM, which is within the physiological range of serum cortisol values (Umland et al. 2002). GR is expressed in two isoforms, namely, GRα and GRβ, which share identical N-termini and are distinguished only by their unique C-terminal ligand-binding domain (Lewis-Tuffin and Cidlowski 2006). GRα binds cortisol, DNA and other transcription factors, thereby modifying the transcriptional activity of target genes (Lu and Cidlowski 2004). On the other hand, GRβ does not bind any ligands and fails to activate transcription, although it forms homodimers that bind DNA. Relative levels of these two GR isoforms in a cell influence the sensitivity of the cell to GC. Higher levels of GRβ lead to GC resistance (Pujols et al. 2001).

## Blood brain barrier

The BBB is a diffusion barrier essential for the normal functioning of the CNS (Ballabh et al. 2004). It is composed of highly specialized endothelial cells, which, together with circulating blood components, pericytes, astrocytes, microglia, neurons and basement membrane form a neurovascular unit (Neuwelt et al. 2008). The capillary endothelial cells that make up the BBB are formed by tight junctions (TJs) that seal paracellular spaces between the endothelial cells thereby restricting paracellular permeability (Wolburg and Lippoldt 2002; Förster 2008). The integral membrane proteins claudin, occludin and junction adhesion molecules make up the TJs, alongside a number of cytoplasmic accessory proteins including zonula occludens (ZO)-1, ZO-2, ZO-3 and cingulin (Ballabh et al. 2004). Junctional protein disintegration plays a major role in BBB breakdown during MS, meningitis, or brain neoplasm (Förster et al. 2008).

## Regulation of BBB by GCs

Many factors regulate the BBB. Changes in the expression of junctional proteins in the endothelial cells through metabolic or cellular mediators play a role in this regulation leading to dynamic changes in BBB properties (Madra et al. 1986; Madara 1988; Schneeberger and Lynch 1992; Citi 1993; Leach et al. 2000; Schweingruber et al. 2011). However, GCs are also important regulators of BBB properties together with other factors such as growth factors or calcium. Treatment of tumor-induced brain edema by GC reduces microvessel permeability. Several studies have demonstrated the molecular mechanisms behind this. For instance, treatment of epithelial cells with dexamethasone, a synthetic GC, influences the differentiation of TJ proteins (Buse et al. 1995). It increases transendothelial electrical resistance (TER) and ZO-1 expression (Singer et al. 1994). In addition, dexamethasone has also been found to influence TJ expression leading to a reduction of permeability in rat (Romero et al. 2003; Calabria et al. 2006), murine (Förster et al. 2005; Weidenfeller et al. 2005), porcine (Hoheisel et al. 1998; Lohmann et al. 2002) and human (Weksler et al. 2005; Förster 2008) endothelial cells. In the advent of a neuroinflammatory response, cell adhesion molecules such as intercellular adhesion molecule-1 (ICAM-1) and vascular cell adhesion molecule-1 (VCAM-1) become upregulated on endothelial cells facilitating lymphocyte extravasation across the BBB (Engelhardt 2006). GCs inhibit BBB disruption thereby reducing leukocyte infiltration into the CNS (Paul and Bolton 1995).

## Genes of vascular endothelium targeted by GCs

### Occludin

The TJ protein occludin was the first integral protein to be identified, initially in the chicken (Furuse et al. 1993) and then in mammals (Anko-Akatsuka et al. 1996). It is composed of four transmembrane domains, two extracellular loops of similar size, three cytoplasmic domains, a short cytoplasmic N-terminus and a long C-terminus (Gonzalez-Mariscal et al. 2003). Although occludin localizes at the TJs, it cannot form TJ strands by itself (Furuse et al. 1996). In addition, the formation of an effective diffusion barrier and epithelial cell polarization are not prevented by the disruption of both occludin alleles in embryonic stem cells (Saitou et al. 1998). Moreover, occludin knock-out mice display well-developed TJs (Saitou et al. 2000).

**Fig. 2:**
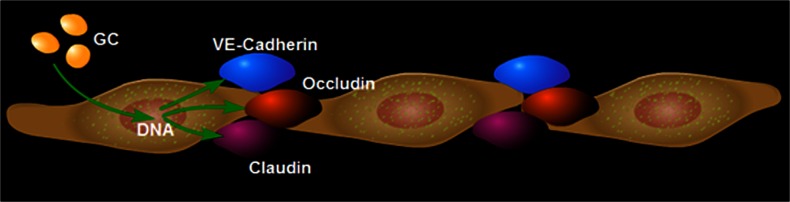
Genes of the microvascular endothelium are targeted by glucocorticoids (*GC*). GC treatment induces the expression of tight junction proteins occludin and claudin-5 and the adherens junction protein vascular endothelium cadherin (*VE-Cadherin*)

Nonetheless, several lines of evidence show that occludin plays an important role at TJs. It contributes to the electrical barrier function of TJs and possibly to the formation of aqueous pores within TJ strands (McCarthy et al. 1996). Moreover, the N-terminal half of occludin has demonstrated its important role for TJ assembly and barrier function maintenance (Bamforth et al. 1999). Overexpression of occludin increases TER in mammalian epithelial cells (Balda et al. 1996). Originally hydrocortisone (HC) was reported to reinforce blood–brain properties in a serum-free cell culture system of cultured porcine cerebral capillary endothelial cells (Hoheisel et al. 1998). Next, GCs were shown to induce a more differentiated BBB phenotype through the modification of TJ structure in cultured rat brain endothelial cells (Romero et al. 2003). Then, the tightening effects of GC treatment was demonstrated in the murine cerebrovascular endothelial cell line cEND (Förster et al. 2005, 2006) and in human hDMEC/D3 cells (Förster 2008). Study of GC effects on the improvement of BBB properties in a murine in vitro system was, for the first time, able to identify occludin as a direct target for GC (Förster et al. 2005). GCs were shown to induce occludin expression (Fig. 2); this was dependent on the GR. A GC response element (GRE) was identified as a GR-binding site within the occludin promoter. This indicated that transactivation of occludin occurred through the GRE (Harke et al. 2008). Moreover, GC treatment antagonized the effects of tumor necrosis factor-α (TNF-α) on occludin expression in endothelial cell lines (Silwedel and Förster 2006).

### Claudins

Despite disruption of both alleles of the occludin gene in embryonic stem (ES) cells, well-developed TJs were found between adjacent epithelial cells. This led to the conclusion that occludin was not necessarily required for TJ formation itself and indicated that other unidentified TJ integral membrane protein(s) could form strand structures without occludin (Saitou et al. 1998). Concomitantly, peptide sequencing led to the discovery of two putative TJ integral proteins that were subsequently named claudin 1 and 2 (Furuse et al. 1998). So far, 24 members of the claudin family have been identified (Tsukita et al. 2001). These proteins show sequence similarity to each other and also bear four putative transmembrane domains but do not show any sequence similarity to occludin (Furuse et al. 1998).

Endothelial cells of the BBB express claudin-1, -3, -5 and -12 (Wolburg and Lippoldt 2002; Krause et al. 2008). Claudin-5 plays an important role in the maintenance of BBB function. Mice that are claudin-5-deficient are born alive but die within a day after birth with no morphological abnormalities. This can be attributed to the integrity of the BBB of these mice being severely decreased, since it is selectively permeable to molecules with a size of < 800 Da (Nitta et al. 2003).

The murine claudin-5 promoter has been cloned and characterized (Burek and Förster 2009). Putative GREs in the promoter sequence have been identified. An increase in claudin-5 promoter activity and mRNA expression occurs after dexamethasone treatment. In addition, increased claudin-5 mRNA and protein expression resulting in higher TER of the endothelial cells has been observed after GC treatment (Fig. 2; Förster et al. 2006; Felinski et al. 2008; Förster 2008; Sadowska et al. 2010). Claudin-5 is also regulated by estrogens on promoter, mRNA and protein levels (Burek et al. 2010).

### Vascular endothelium cadherin

Together with TJs, adherens junctions (AJs) maintain the restrictiveness of the barrier in endothelial cells (Fig. 2). The most important AJ protein is vascular endothelial cadherin (VE-cadherin), which is exclusively expressed in vessels in which it regulates Ca^2+^-mediated adhesion (Dejana et al. 1999; Dejana 2004; Gumbiner 2005; Gavard 2009). Deletion of VE-cadherin in mice produces massive vascular effects leading to early embryonic death. On the other hand, loss of its function provokes a hyperpermeability in adults (Carmeliet et al. 1999; Crosby et al. 2005).

Treatment of endothelial cells with dexamethasone increases VE-cadherin protein levels. Transcriptional activation of the VE-cadherin promoter by dexamethasone, however, does not point to direct GC-mediated VE-cadherin gene induction (Blecharz et al. 2008). Although TJs and AJs are formed by different molecules, they are functionally and structurally linked. VE-cadherin at AJs upregulates the gene encoding claudin-5 via the release of the inhibitory activity of the forkhead box factor FoxO1 and the Tcf-4-beta-catenin transcriptional repressor complex (Taddei et al. 2008).

## GC therapeutic strategies and their limitations

**Fig. 3:**
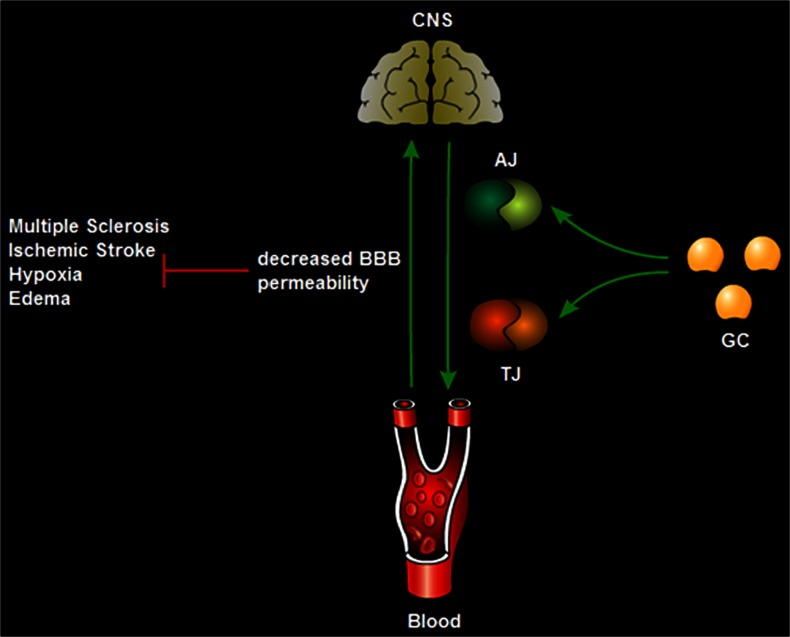
Most diseases and disorders of the central nervous system (*CNS*) involve the disruption of the blood brain barrier (*BBB*), decreasing its permeability. Administration of glucocorticoids (*GC*) induces the expression of adherens junctions (*AJ*) and tight junctions (*TJ*) leading to increased barrier tightness

The BBB is compromised in many CNS disorders. To function normally, the CNS needs to maintain its microenvironment; this is performed by the BBB via its regulation of molecule transport between the blood and the brain. Since TJ and AJ proteins contribute to endothelial cell barrier restrictiveness, the effects of GCs toward upregulation in the expression of these proteins leading to a tightening of the barrier are used to advantage for therapeutic purposes against many diseases of the CNS including MS, human immunodeficiency (HIV)-1-associated dementia, CNS vasculitides, stroke, Alzheimer’s disease and cerebral malaria (Fig. 3).

### Multiple sclerosis

MS, a neuroinflammatory disorder characterized by infiltrating autoreactive T-cells in the CNS, is the most prevalent chronic autoimmune disease in the western world (Noseworthy et al. 2000; Sospedra and Martin 2005). Most patients suffer from a relapsing-remitting form of the disease whose exacerbations are initiated by BBB breakdown and subsequent tissue damage. Because of the infiltration of activated immune effector cells into the brain, an inflammatory cascade involving leukocytes and humoral components arises (Steinman 2001; Engelhardt 2006). Even though GC treatment has little effect on the long-term prognosis of MS, high dosage GC therapy is an approved first line treatment for MS relapses (Milligan et al. 1987; Reichhardt 2004).

For more than 70 years, experimental autoimmune encephalomyelitis (EAE) has served as a model for MS (Gold et al. 2006). In using this model, together with studies of MS patient, multiple beneficial effects of GC treatment have been established. GCs have anti-inflammatory and immunosuppressive activities and can induce apoptosis. Their induction of apoptosis in T-cells leads to the activation of caspases, release of cathepsin B from lysosomes and increased H_2_O_2_ levels (Screpanti et al. 1989; Gold et al. 2006). GCs inhibit the disruption of the BBB and hence reduce leukocyte infiltration into the CNS (Paul and Bolton 1995). Direct effects of GCs on the BBB have been demonstrated in vitro (Tischner and Reichardt 2007). For instance, in cultured brain endothelial cells, dexamethasone induces pore reduction and F-actin concentration on the cell periphery and increases ZO-1 and occludin expression (Romero et al. 2003; Förster et al. 2005, 2006). In addition, the administration of dexamethasone and HC preserves the functional integrity of TJs and AJs under pro-inflammatory conditions by maintaining the levels of TJ components such as occludin, claudin-1, claudin-12, ZO-1 and VE-cadherin. However, dexamethasone effects on claudin-5 are negligible (Förster et al. 2007; Blecharz et al. 2008).

ICAM-1 and VCAM-1 are expressed by endothelial cells of the BBB in response to pro-inflammatory cytokines such as interferon-γ and TNFα. Treatment with GCs reduces the expression of these adhesion molecules (Gelati et al. 2000; Sloka and Stefanelli 2005). Accordingly, integrins on encephalitogenic T-cells interact with these adhesion molecules, which are required for the extravasation of additional effector T-cells into the CNS (Engelhardt 2006). Production of interleukin-8 by monocytes, which also contributes to BBB disruption through leukocyte recruitment to the CNS, is also decreased in MS patients following GC treatment (Mirowska-Guzel et al. 2006).

The opening of the BBB as promoted by cytokines has been shown to occur because of TJ protein degradation and decreased synthesis. This results in compromised junctional integrity (Harkness et al. 2000; Chang and Werb 2001; Silwedel and Förster 2006; Yang et al. 2007). TJ degradation in neuroinflammatory conditions such as MS results from the effects of matrix metalloproteinases (MMPs) in BBB disruption. MMP-9 (gelatinase B) is increased in cerebrospinal fluid levels in MS patients. An important regulator of MMP activity is via binding to a family of homologous proteins referred to as tissue inhibitors of MMPs (TIMPs; Brew et al. 2000). All four known TIMPS have been demonstrated in the BBB endothelium (Hartmann et al. 2009). In MS patients, TIMP levels are reduced in comparison with controls. This suggests an imbalance in MMP-9/TIMP ratios (Avolio et al. 2005). GC treatment reduces levels of MMP-9, based on the GC-mediated activation of the TIMP-1 gene and has been shown to lead to an upregulation of TIMP-1 in the murine cerebral vascular endothelial cell line, cEND (Förster et al. 2007). In contrast, HC has been revealed selectively to upregulate TIMP-3, whereas TIMP-1, TIMP-2 and TIMP-4 are downregulated at the mRNA-level in endothelial cells of cerebral capilllaries in porcine brain. This effect can be completely reversed by the GR inhibitor mifepristone. The application of HC leads to a strong enrichment of TIMP-3 in the endothelial cell membrane (Hartmann et al. 2009). Moreover, the downregulation of claudin-5 and occludin protein and mRNA levels occurs together with MMP-9 expression upregulation after incubation of the murine microvascular endothelial cells, cEND, with serum from MS patients (Blecharz et al. 2010).

### Ischemic stroke

GCs are known to stabilize the BBB in many inflammatory CNS disorders and even to decrease edema formation. However, in some other cases of CNS disease, it tends to aggravate the condition rather than diminish the negative effects. For instance, when GC treatment is applied to acute ischemic stroke, it is ineffective or even harmful (Saul et al. 1981; Norris and Hachinski 1986; De Reuck et al. 1988; Kumar et al. 1989; Norris 2004; Poungvarin 2004; Roberts et al. 2004). This is attributed to GC insensitivity at the hypoxic BBB. In the cultured murine brain microvascular endothelial cells, cEND, O_2_/glucose deprivation reduces TJ protein expression and TER. Treatment with dexamethasone fails to reverse these effects during hypoxia. Apparently, proteasome-dependent degradation of the GR impairs GR transactivation, in turn preventing physiological GC activity. Inhibition of the proteasome with Bortezomib, however, fully restores the BBB stabilizing properties of GC during O_2_/glucose deprivation (Kleinschnitz et al. 2011).

### Effects of ubiquitination on GC response during hypoxia

Post-translational modifications of the GR include phosphorylation under various conditions by cyclin-dependent kinases and mitogen-activated protein kinases (MAPKs), sumoylation and ubiquitination (Tischner and Reichardt 2007). Ubiquitination determines protein longevity in cells and plays major roles in signaling (Ernst and Sidhu 2013) being a form of post-translational modification that targets a protein for rapid degradation by the proteasome (Meller 2009). Excessive proteosomal GR degradation in response to long-term GC exposure has been shown to lead to GC insensitivity in the vascular endothelium (Förster et al. 2006). An endothelial GR protein has been identified as a potential proteasome substrate after hypoxia (Kleinschnitz et al. 2011).

GC responses are dependent on the post-translational modification and degradation of GR to which GC binds to form a complex and begin its activity. The ubiquitin-proteosome system plays a major part in this modification and degradation. Degradation of the proteasome by nuclear receptors is a physiological process necessary to terminate transcriptional activity after ligand binding. It can, however, also restrict transcriptional signaling by steroids under certain pathophysiological conditions thereby compromising steroid function (Meller 2009). In an in vitro BBB model subjected to O_2_/glucose deprivation, degradation of the GR in a proteasome-dependent manner leading to impaired GC sensitivity has been observed. When the proteasome is inhibited, the responsivity of the BBB to GC during hypoxia is restored. When proteosomal inhibition is combined with GC treatment, edema formation is attenuated, as are neurological deficits after transient middle cerebral artery occlusion (tMCAO) in mice, which is an established in vivo model of hypoxic BBB damage (Kleinschnitz et al. 2011).

In addition, studies have shown that cell-death-mediating proteins are rapidly degraded by the ubiquitin-proteosome system. For instance, some pro-apoptotic proteins such as Bim and Puma have been identified as direct GR targets (Erlacher et al. 2005; Wang et al. 2006). Therefore, a better understanding of the mechanisms by which this occurs might prove to be useful for therapy during ischemia and other brain injuries.

### Action of GC-based combination therapy with proteosomal inhibitors against edema

Although GCs diminish edema formation in neuroinflammatory diseases such as acute MS lesions and in certain brain tumors, they are ineffective or even harmful in brain disorders such as acute ischemic stroke or traumatic brain injury (TBI; Kumar et al. 1989). Stroke and other forms of ischemic brain injury result in excessive edema formation in the brain leading to morbidity and mortality (Kahle et al. 2009). Therefore, the finding that GCs can diminish edema in some neuroinflammatory diseases but not in ischemia or TBI is unfortunate.

Degradation of GR occurs during O_2_/glucose deprivation in cEND cells and in the tMCAO model of stroke (Kleinschnitz et al. 2011). However, the use of pharmacological proteasome inhibitors is able to prevent it. GR transactivation by GC binding has been found to be maintained through the overexpression of a GR mutant resistant to proteasomal degradation (GR-K426A mutant; Wallace and Cidlowski 2001). These findings have led to the development of a combined treatment strategy by using GCs combined with inhibitors of the proteasome. This novel therapy effectively and significantly reduces edema formation. Furthermore, it significantly ameliorates neurological performance in mouse models of stroke and TBI (Kleinschnitz et al. 2011).

## Concluding remarks

The ability of GCs to improve barrier properties and induce the expression of TJ proteins occludin and claudin-5 and AJ protein VE-cadherin demonstrates their positive effects toward the BBB. Even though high dosage administration of GCs has been found to be ineffective or even harmful in brain disorders such stroke or TBI, novel strategies such as combination therapy of GCs and proteasome inhibitors have proven effective in the prevention of BBB disruption, edema formation and neuronal damage upon brain injury both in vivo and in vitro. Therefore, further studies concerning the GR-mediated regulation of BBB permeability are needed for the development of more and better therapeutic strategies with GCs.

Currently, the establishment of an in vitro human stroke model based on human brain capillary endothelial cells in our working group together with cooperating partners is underway. The model is in the process of being tested, verified and strengthened for use in various experimental procedures. Once this model is established, it should prove highly beneficial in medical and biological research.
